# Supplementary material for: Non-Covalent Linkage of Helper Functions to Dumbbell-Shaped DNA Vectors for Targeted Delivery
Source: Pharmaceutics. 2023 Jan 21;15(2):370. doi: 10.3390/pharmaceutics15020370 (PMC9962178; doi:10.3390/pharmaceutics15020370)
Supplement: Supplementary file 1 [file pharmaceutics-15-00370-s001.zip › pharmaceutics-2066798-supplementary.pdf]

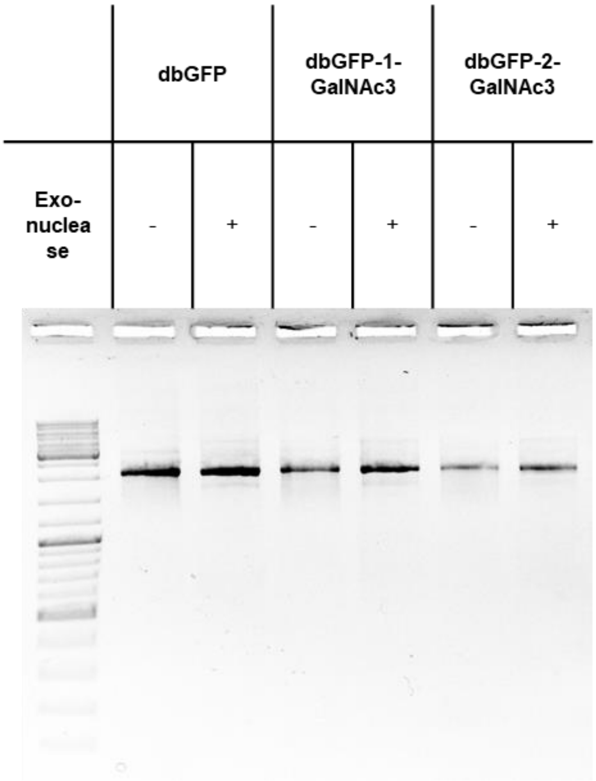

Supplementary Figure S1 Dumbbell vectors are exonuclease resistant. Analytical 1% agarose gel analysing MaxGFP expressing dumbbell vectors before and after exonuclease treatment.

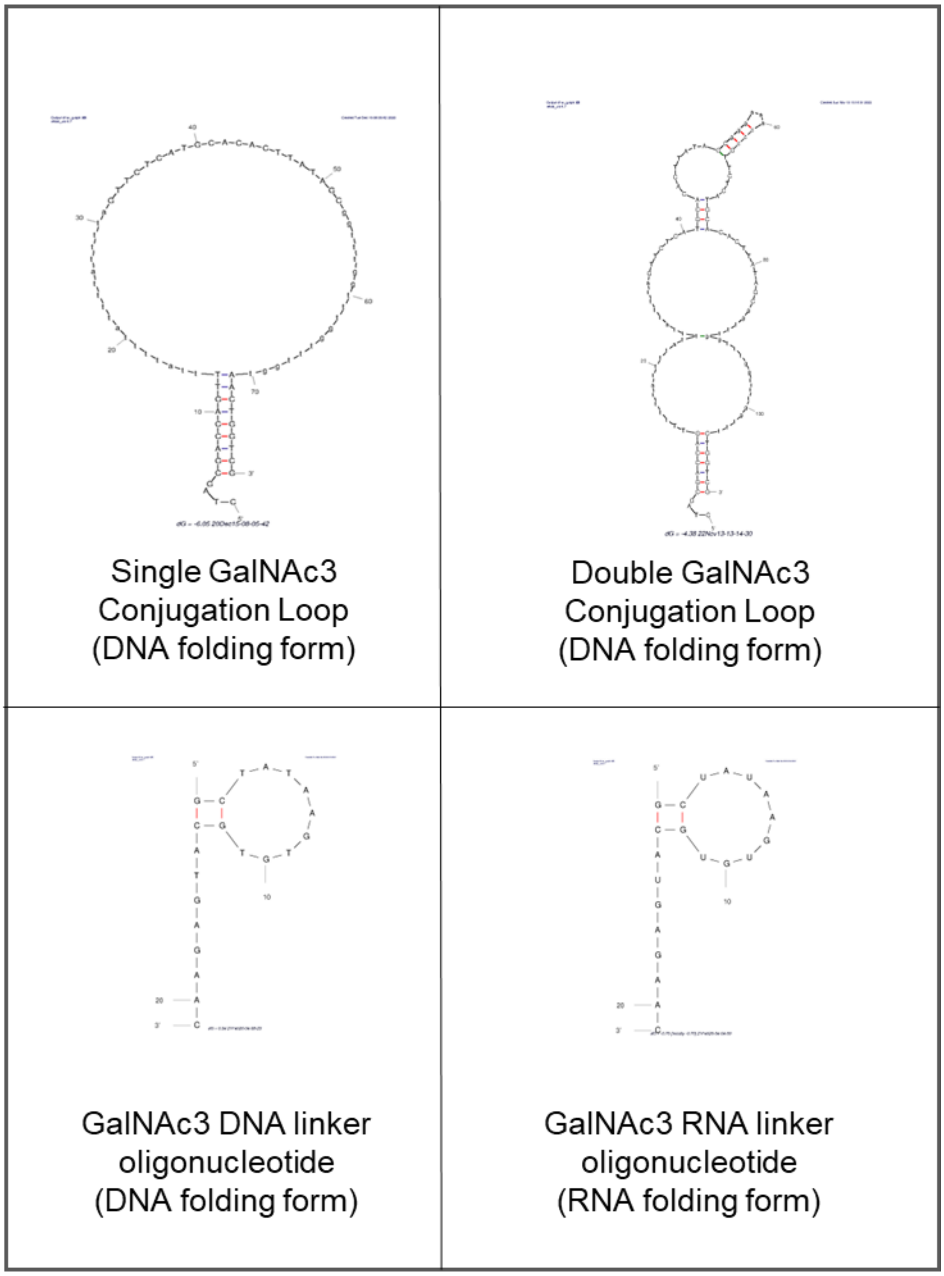

Supplementary Figure S2 Minimum free energy secondary structures. Minimum free energy secondary structures of single and double GalNAc3 conjugation loops (upper panel) and of GalNAc3 DNA and RNA linker oligonucleotides (lower panel) as predicted by mfold's DNA or RNA folding forms [20].

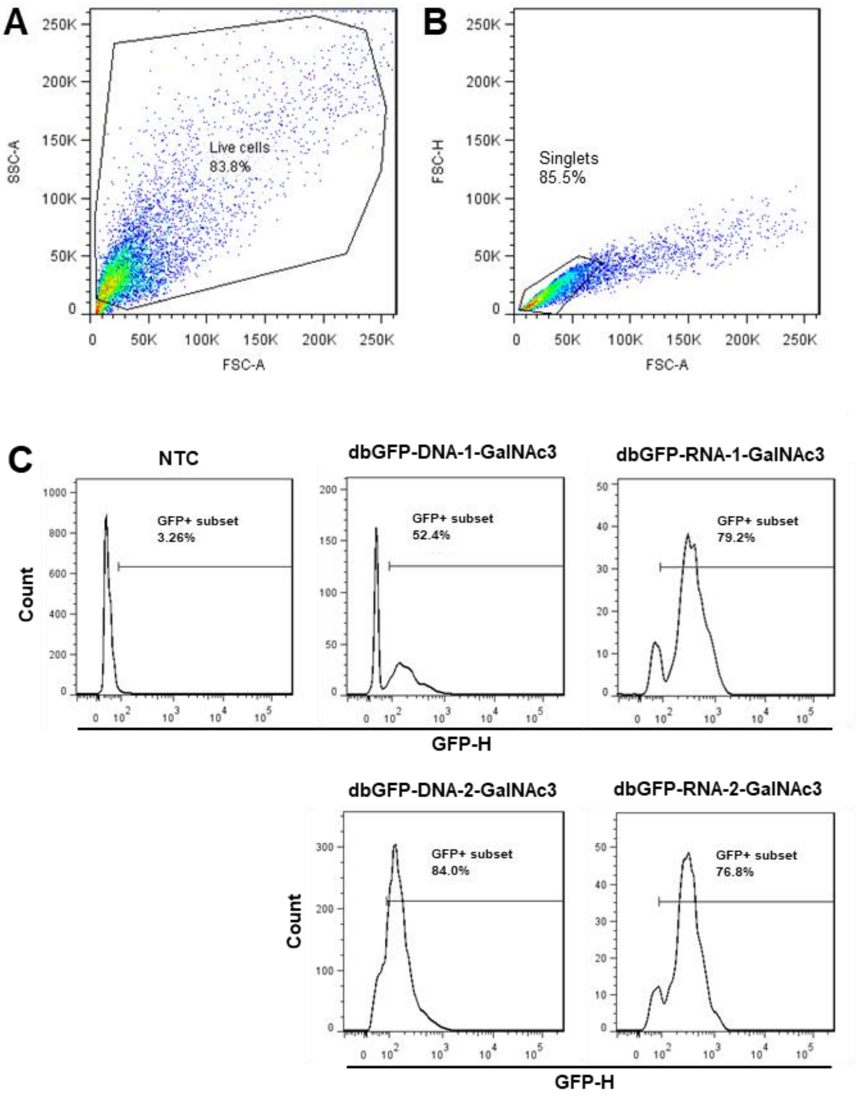

Supplementary Figure S3 Flow cytometry analyses of HepG2 cells transfected with MaxGFP dumbbell-GalNAc3-conjugates. **A**, Representative 2-D scatter plot gating for live HepG2 cells. **B**, Representative 2-D scatter plot gating for HepG2 cell singlets. **C**, Representative histograms of flow cytometry analyses of HepG2 cells exposed for 48 hours to dbGFP-GalNAc3 conjugates added to the cell culture medium. NTC: No-transfection control; dbGFP-DNA-1-GalNAc3: MaxGFP expressing dumbbell conjugated with 1 GalNAc3 residue via a DNA linker; dbGFP-RNA-1-GalNAc3: MaxGFP expressing dumbbell conjugated with 1 GalNAc3 residue via a RNA linker: dbGFP-DNA-2-GalNAc3: MaxGFP expressing dumbbell conjugated with 2 GalNAc3 residues via a DNA linker: dbGFP-RNA-2-GalNAc3: MaxGFP expressing dumbbell conjugated with 2 GalNAc3 residues via a RNA linker.
